# Application of Deep Learning in College Physical Education Design under Flipped Classroom

**DOI:** 10.1155/2022/7368771

**Published:** 2022-09-16

**Authors:** Jun Huang, Dian Yu

**Affiliations:** School of Competitive Sports, Shandong Sport University, Rizhao 276827, Shandong, China

## Abstract

With the development of information technology, teaching reform has also undergone major changes. The traditional college physical education teaching method cannot meet the needs of the majority of students, and the physical education teaching mode continues to be reformed. Microcourse is the most intuitive form of deep integration of information technology and physical education. From the perspective of the flipped classroom (FC), the physical education model has gradually changed from teacher centered to student centered. Deep learning (DL) emphasizes that learners have the ability to actively construct knowledge, effectively transfer knowledge, and solve real problems. This design applies DL and convolutional neural network to the teaching design of physical gymnastics in colleges and universities. The application of the DL teaching model based on FC in the microcourse teaching of gymnastics in colleges and universities is studied and evaluated. The results show that the current utilization of microcourse teaching resources is too low. Interest-oriented teaching microcourses cannot improve students' interests. The proportion of students who are interested is relatively small, and more than 50% of students are not interested. Teachers generally believe that the current gymnastics microcourse needs further optimization and improvement. The poor quality of microvideos and the lack of supervision and reward mechanism in the course are the main reasons for the insufficient students' interest. The complexity of the videos and the liveliness of the discussions are the main problems of low resource utilization. The student's interest in learning is greatly improved after the application of the designed model, and the proportion increases to 82.4%. The effect on ordinary college students is the most obvious, and the effect of microvideo learning has been significantly promoted. Design mode has the most obvious improvement in improving learning efficiency and autonomous learning ability. The improvement of learning ability has increased from 18% to 72%, and the improvement of learning efficiency has increased from 39% to 82%. Meanwhile, students' interest in learning is stimulated, and the utilization of resources is improved.

## 1. Introduction

With social progress and technological development, China's educational reform level has also been greatly improved. Physical education has also been valued by colleges and all sectors of society, but the final teaching effect is not very obvious. Deepening teaching reform is crucial. The reform of teaching methods is an important manifestation of teaching reform. New ideas have been brought to the innovation of physical education teaching in colleges and universities with the continuous deepening of the flipped classroom (FC) education model and the gradual use of information-based teaching methods. In addition, it brings the possibility to improve the quality of physical education teaching in colleges and universities. The FC has played a huge role in promoting the reform of physical education teaching in colleges and universities [1].

In today's society, scholars have also conducted various researches on physical education in colleges and universities. Some researchers have deeply discussed how to apply FC to taekwondo teaching in colleges and universities. They have studied whether FC can help improve the taekwondo skills of college students through educational practice and questionnaire methods [2]. Hinojo Lucena et al. used the method of literature analysis and case analysis to take “FC” as the research object and focused on analyzing its application strategies in physical education [3]. Julia and Marco analyzed the basic theoretical knowledge of public sports as the teaching content of the public sports massive open online courses (MOOCs) using the literature method and questionnaire survey method. They compared the implementation effect of MOOC technology [4]. Fang et al. combined the FC teaching mode with physical education teaching based on knowledge construction and made an objective teaching evaluation of this teaching mode [5]. Liao proposed the practical application of the FC model in the teaching of sports table tennis in colleges and universities under the background of MOOCs [6]. Students are mostly in a passive acceptance state in the traditional lecture-style teaching process. Simple memory and repetitive training hinder the transfer of learner knowledge [7]. Deep learning (DL) advocates for learners to actively apply what they have learned to solve complex problems in reality. It helps to enhance critical thinking and knowledge construction [8]. Tufail et al. applied a DL 3D convolutional neural network (CNN) architecture to disease diagnosis [9]. Tufail et al. conducted a taxonomic study of the endangered jujube species in the European habitat ecosystem based on a DL architecture model [10]. Khan et al. applied DL models to the multiclass classification problem of modulated signals in intelligent communication systems [11]. Tufail et al. applied DL to help determine optimal model designs for cancer diagnosis and prediction tasks [12]. The integration of DL and teaching has become a research hotspot in education. Shuo and Ming [13] combined DL with smart classrooms and designed a “3 + 1” smart teaching model. Jan et al. [14] summarized the process of educational big data mining based on the characteristics of educational big data mining under DL. Through literature research, it is found that DL models are widely studied in medical care, ecosystems, and communications and are also infiltrated in teaching. Research on physical education teaching in colleges and universities is scarce.

Based on this, under the concept of FC, this study innovatively combines the CNN model in the DL algorithm with the teaching design of college sports gymnastics microcourse. The application of the teaching mode is investigated through a questionnaire survey, and the application effect is further analyzed. This design aims to improve the teaching effect of gymnastics microcourse, stimulate learning interest, and promote the wide application of microcourse resources by changing the gymnastics teaching mode. Although there are some limitations, it is expected that the improvement of the design concept and teaching mode can provide a reference for the subsequent reform and construction of college physical education.

## 2. Methods

### 2.1. FC

FC is the product of the combination of network and face-to-face teaching platforms and is a new teaching model formed under the support of modern information technology [15]. This learning model can be widely applied to various educational and teaching processes. FC frees teachers from tedious teaching procedures, leaving time to focus on students. Teachers establish a close relationship with students to enhance students' autonomous learning motivation [16]. FC innovates the traditional education model, and the teaching model has many characteristics, such as innovative teaching mode, student-centered differentiated teaching mode, personalized blended learning mode, and dynamic classroom [17]. In this education model, students have changed from passive to active, and their learning methods have also changed to a model of preclass learning and classroom inquiry. The teaching method is not limited by the location, which is convenient for students to learn [18]. Taking student learning as the center can continuously stimulate students' subject consciousness and cognitive ability. Besides, it stimulates the creativity and participation of students.

### 2.2. DL Theory

DL is a machine learning method with multiple neural network layers, which has a wide range of applications in many fields. DL has been applied in graph analysis, data processing, and public data prediction and has powerful feature information extraction and learning capabilities [19, 20]. Learners can critically learn new knowledge and update existing cognitive structures while transferring knowledge to new situations [21]. DL extracts features layer by layer by mining the underlying feature distribution of the data and transforms low-level feature data into high-dimensional features [22]. DL consists of three layers, namely, input layer, hidden layer, and output layer.

DL can use standardization, normalization, and other operations for data preprocessing to make the data meet the training needs [23]. Normalized data are handled by scaling with the two maxima:

(1) $$\begin{array}{c} X_{i}=\frac{X_{i}-\min\left(X\right)}{\max\left(X\right)-\min\left(X\right)}\text{.} \end{array}$$

Normalization is a transformation for each feature. In equation (1), max(*X*) represents the maximum value of a feature and min(*X*) represents the minimum value of a feature. The value range of all data transformations is [0, 1].

Normalization is to make the sample size have a uniform standard when calculating the similarity by dot product or kernel function. Each sample is transformed, and the original data are transformed into a unit vector. The conversion process is shown as follows:

(2) $$\begin{array}{c} x=\frac{x}{\sqrt{\overset{n}{\underset{j}{\sum }}\;X{\left[j\right]}^{2}}}\text{.} \end{array}$$

In equation (2), *n* is the number of sample features and *X*[*j*] is the *j*th feature of sample *x*. The function of normalization is to make the sample vectors have a uniform standard when similarity is calculated by dot multiplication or kernel function.

**Figure 1:**
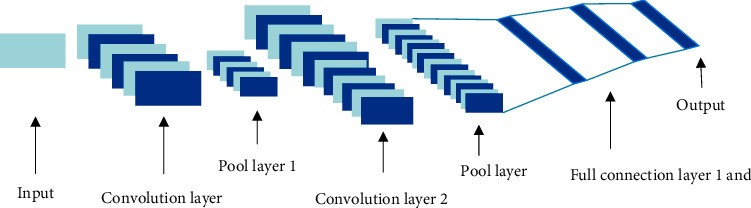
DL model.

CNN is one of the typical algorithms in DL. Partial connections are used between the internal layers of the CNN. Weight sharing can be achieved between neurons in the same layer. CNN model parameters are reduced, which has obvious advantages in speech recognition and image recognition tasks. A CNN consists of an input layer, a convolutional layer, a pooling layer, a fully connected layer, and an output layer [24]. The DL model is shown in Figure 1.

From Figure 1, the input layer is responsible for data input and further preprocesses the input data to pave the way for the next step of CNN convergence. Convolutional layers use convolution to extract multiple features of input data or images. When the convolution kernel performs convolution processing, it scans the input data according to step size, performs matrix multiplication and summation in the convolution kernel, and adds the deviation:

(3) $$\begin{array}{c} \begin{array}{c} Z^{l+1}\left(i,j\right)=\left[Z^{l}⊗w^{l+1}\right]\left(i,j\right)+b\\ =\overset{K_{l}}{\underset{k=1}{\sum }}\;\sum_{x=1}^{f}\;\overset{f}{\underset{y=1}{\sum }}\;\left[Z_{k}^{l}\left(s_{0}i+x,s_{0}i+y\right)w_{k}^{l+1}\left(x,y\right)\right]+b\text{.} \end{array} \end{array}$$

In equation (3), *b* is the deviation. *Z*^*l*^ and *Z*^*l*+1^ are the convolution input and output of the *l*+1th layer. *Z*(*i*, *j*) is the pixel of the feature map, and *K* is the number of channels of the feature map. *f* and *s*_0_ are the size of the convolution kernel and the convolution step size, respectively.

The value range of pixel (*i*, *j*) is shown in the following equation:

(4) $$\begin{array}{c} \left(i,j\right)\in \left\{0,1,\ldots ,L_{l+1}\right\}\text{,} \end{array}$$

(5) $$\begin{array}{c} L_{l+1}=\frac{L_{1}+2p-f}{s_{0}}+1\text{.} \end{array}$$

In equations (4) and (5), *p* is padding and *L*_*l*+1_ is the size of *Z*^*l*+1^.

The pooling layer compresses the convolutional features and uses the pooling function in the pooling layer to count the value of each area in the feature map, including pooling size, step size, and padding. The pooling method is shown in the following equation:

(6) $$\begin{array}{c} A_{k}^{l}\left(i,j\right)={\left[\overset{f}{\underset{x=1}{\sum }}\;\overset{f}{\underset{y=1}{\sum }}\;A_{k}^{l}{\left(s_{0}i+x,s_{0}i+y\right)}^{p}\right]}^{1/P}\text{.} \end{array}$$

In equation (6), the pixel (*i*, *j*) is the same as the convolutional layer and *p* is the pooling parameter.

After the image passes through the pooling layer, it is flattened and passed to the fully connected layer. At this time, the image loses its spatial characteristics and is expanded into the form of a vector.

## 3. Application of the DL Teaching Mode Based on FC in Microcourse Teaching of Physical Gymnastics in Colleges and Universities

### 3.1. Microcourse

**Figure 2:**
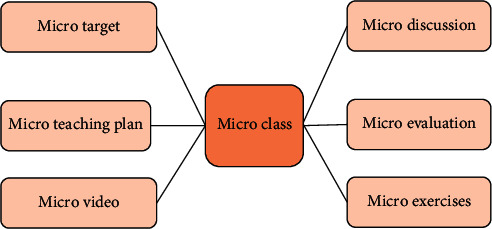
Microcourse teaching resources.

“Physical education professional gymnastics microclass” focuses on the teaching goals and difficulties of gymnastics courses. It takes teaching videos as the main carrier, emphasizes active learning, and carries out online network teaching activities in network media. In general, microcourses are presented in the form of microvideos [25]. As a kind of course resource, the specific content of the microcourse is shown in Figure 2.

In Figure 2, the teaching resources of microcourses mainly include microtargets, microvideos, microteaching plans, microexercises, microdiscussions, and microevaluations. Various teaching resources of microcourse serve the teaching process and students' autonomous learning process. The teaching application mode of microcourse is divided into three types, namely, flipped teaching, classroom differentiated teaching, and after-school tutoring and answering application mode. Among them, the flipped teaching application mode is the most commonly used teaching mode in microcourses. The microcourses under its teaching application mode can be placed before the class or during the class, forming a model of “learning first and teaching later.”

**Figure 3:**
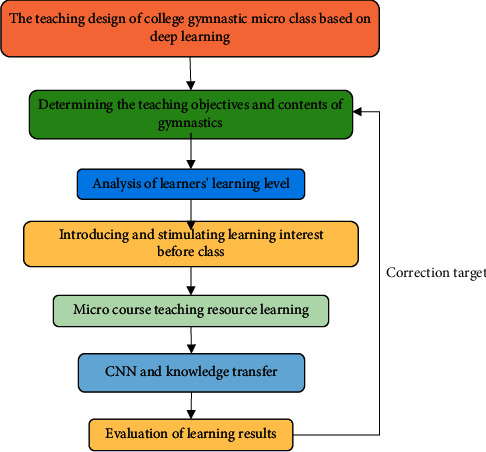
Teaching design of college sports gymnastics microcourse based on DL.

Gymnastics is a compulsory course for physical education and training, covering the characteristics of physical education and being representative. Physical education professional gymnastics microcourse is taken as the research object. The microcourse teaching mode of gymnastics in colleges and universities is designed using the in-depth learning route. The design content includes seven parts, which are determining teaching objectives and content, preassessment and analysis, interest-oriented stimulation of students' interest in learning, learning with microcourse resources, learning task transfer, learning effect evaluation, and learning correction [26]. The specific design route is demonstrated in Figure 3.

The design of learning objectives and content emphasizes that teachers conduct an in-depth study of course requirements, and teachers are guided to determine teaching objectives. The preassessment analysis focuses on preassessing the learner's level. Preassessment is a prerequisite for inducing DL. It is very important to create a learning cultural atmosphere to stimulate students' learning interests, so students can actively participate in real learning situations. Learners update and recognize based on their original knowledge in the process of learning. It is necessary to stimulate the students' prior knowledge and activate the neural network of the brain before knowledge is transferred. Knowledge is processed through DL knowledge and evaluated. Teaching objectives are continuously revised through evaluation feedback.

### 3.2. Research Methods

Expert interviews and questionnaires are combined here. Teachers in charge of sports microcourses are interviewed to clearly understand the current teaching resources of microcourses for physical education majors. Relevant experts in this field are visited to discuss development strategies. Further related research is carried out using the questionnaire survey to understand the actual situation.

### 3.3. Questionnaire Design and Distribution

The subjects of the questionnaire are students of physical education in three different colleges and universities of physical education in Shandong and professional teachers engaged in microcourse teaching of physical education. The three colleges and universities are 985 colleges, 211 colleges, and general colleges. The questionnaire design consists of three parts: the first part is the analysis of the current demand and situation of gymnastics microcourse teaching resources for students majoring in physical education. The second part is a questionnaire for teachers to optimize the teaching resources of the gymnastics microcourse. The third part is a questionnaire on the application effect of the new teaching model of gymnastics microcourse by students majoring in physical education.

Questionnaires are distributed mainly through the Questionnaire Star platform. The distribution and recovery of questionnaires for students and teachers are listed in Table 1.

The first part of the questionnaire survey is for students, with a recovery rate of 92.31% and an effective rate of 95.83%. The second part of the questionnaire survey is for teachers, the recovery rate is 100%, and the effective rate is 91.67%. The third part investigates the teaching effect, the recovery rate is 96%, and the effective rate is 93.75%.

### 3.4. Questionnaire Validity Evaluation

Ten relevant experts are solicited to evaluate the validity of the three parts of the questionnaire. The results are listed in Table 2.

On the whole, the design of the questionnaire meets the basic survey requirements, and the content of the questionnaire is perfect.

### 3.5. Questionnaire Reliability

A second questionnaire survey is conducted on a part of the same batch of respondents, with an interval of one month. The data are processed using statistical product and service solutions. The correlation is calculated using the Cronbach coefficient equation. The correlation coefficients are *R*^1^ = 0.81, *R*^2^ = 0.82, and *R*^3^ = 0.80 through statistical calculation. The results indicate that the questionnaire meets the statistical test standard and has high reliability. Excel is used to analyze the data. The Cronbach coefficient equation can be expressed as follows:

(7) $$\begin{array}{c} \alpha =\left(\frac{n}{n-1}\right)\left(1-\sum \frac{Si}{St}\right)\text{.} \end{array}$$

In equation (7), *α* is the reliability coefficient and *n* is the number of test items. *Si* is the variance of each subject's score for each question and *St* is the variance of the total score obtained by all subjects.

## 4. Results and Discussion

### 4.1. An Analysis of Interest-Oriented Teaching Philosophy

**Figure 4:**
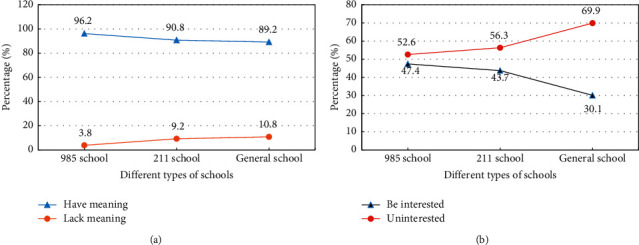
Analysis of the current stage of teaching design concept based on interest. (a) Teachers' attitude towards teaching concepts. (b) Students' interest in microcourses.

An interest-oriented survey of current teaching design concepts is conducted on teachers and students respectively. Teachers' attitudes towards teaching concepts and students' interests in different schools are analyzed. The results are shown in Figure 4.

From Figure 4, most teachers prefer an interest-oriented teaching philosophy. The proportion of ordinary colleges and universities decreases slightly, accounting for 89.2%. The highest proportion is 985 colleges. A few teachers think that the current teaching concept is meaningless. Overall, the interest-based teaching philosophy makes sense. In the evaluation of students' interest in learning, the proportion of interested students is relatively small, and the proportion of students who are not interested exceeds 50%. Among the uninterested students, ordinary colleges accounted for 69.9%, 211 colleges accounted for 56.3%, and 985 colleges accounted for 52.6%.2

### 4.2. Teachers' Self-Evaluation Analysis of Microcourses

**Figure 5:**
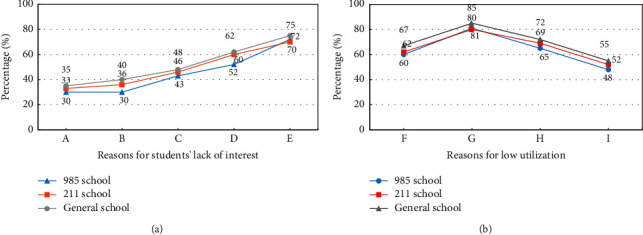
Teachers' self-evaluation analysis of microcourses. (a) Evaluation of the reasons for the lack of students' interest. (b) Evaluation of the low utilization rate of microcourse teaching resources.

Through the questionnaire, teachers' self-evaluation of sports microlecture is mainly reflected in two aspects, namely, the evaluation of the reasons for the lack of students' interest and the evaluation of the low utilization rate of microlectures teaching resources.  Figure 5 shows the specific content of the evaluation.

In Figure 5, A stands for unaccustomed learning style and B stands for lack of initiative. C stands for a single type of homework question and D stands for the lack of supervision and reward mechanism for learning. E means that the quality of the microvideo is not high and F means that the teaching courseware is cumbersome. G means that the microvideo is too long, H means that the discussion area is not active, and I means that the assessment method is single.  Figure 5 shows that there are serious problems with the video quality of microlectures. At least 70% of the students responds that there is a problem with the sound quality of the picture, which is also the main reason for disinterest, followed by the lack of supervision and reward mechanisms. Almost 50% of the students are dissatisfied, and the proportion of students who are not used to learning methods is low. The level of student dissatisfaction is greater than that of 211 colleges and 985 colleges. The main reason for the low resource utilization in Figure 5(b) is that the microvideo is too long, with a proportion exceeding 80%, followed by the inactive discussion area and cumbersome teaching courseware. The last is a single test method. Educational resources need to be further developed, and the quality of teaching should be improved with the goal of learning interest.

## 5. Evaluation of the Application Effect of the DL Teaching Model in the Microcourse Teaching of Physical Gymnastics in Colleges and Universities

### 5.1. Comparative Analysis of Students' Interest in Gymnastics Microcourses

**Figure 6:**
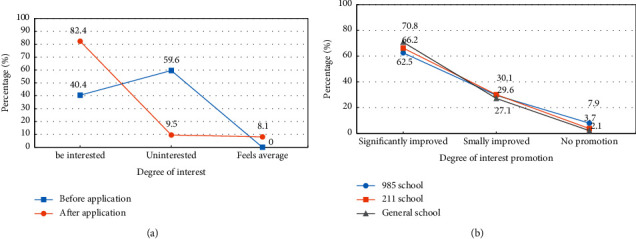
Comparison of increased interest in gymnastics microcourses. (a) Analysis of interest degree before and after application. (b) Analysis of interest promotion degree after the application.

The students' interest in gymnastics microlectures before and after the application of the DL teaching model is analyzed, and the improvement of interest after the application is statistically compared. The results are shown in Figure 6.

From Figure 6(a), the proportion of students who are not interested before the application is large, and the proportion of students who are not interested after the application has decreased significantly. The interest rate increases to 82.4%. After the application, there are still very few students who think it is average, accounting for only 8.1%. From Figure 6(b), the proportion of obvious interest enhancement is more than 60%. The proportion of small interest increases is about 30%. In addition, less than 10% of students think there is no improvement. The effect is most obvious for students in ordinary colleges and universities, with a significant increase ratio of 70.8%, and a proportion of 2.1% who believe that there is no improvement. It is concluded that the design model can greatly improve the learning interest of students in gymnastics microlectures.

### 5.2. Designed Model Application Effect Evaluation and Analysis

**Figure 7:**
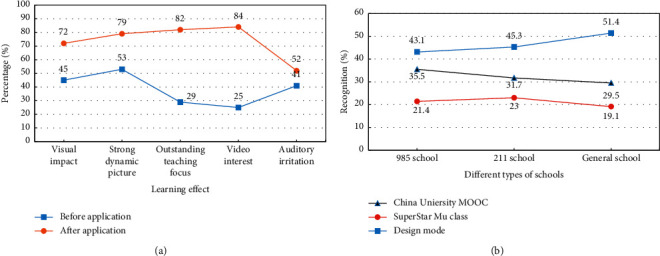
Comparative analysis of microvideo learning effects. (a) Microvideo learning effect analysis. (b) Comparative analysis of learning methods.

The degree of recognition of the students' microvideo learning effect after the model is applied for a year is analyzed. The design model is compared with the teaching methods of MOOC and Chaoxing MOOC in Chinese universities. The results are shown in Figure 7.

From Figure 7, after the application of the design model, the teaching focus has been significantly improved, and the recognition rate has increased from 29% to 82%. Video interest has also been significantly improved, with approval from 25% to 84%. The visual impact and the dynamism of the picture have also been improved to a certain extent. The hearing effect has not improved much, from 41% to 52%. Overall, the design model can effectively improve the learning effect of microvideo. Student recognition has increased significantly. From Figure 7(b), the design model recognition is higher than that of Chinese university MOOCs and Chaoxing MOOCs, with the highest recognition.

### 5.3. Analysis of the Help of Designed Models in Learning

**Figure 8:**
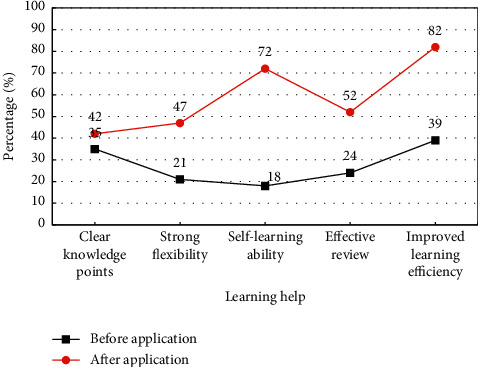
Analysis of the help of designed models in learning.

The help of the DL design model to students' learning after the application is analyzed, and it is compared with before the application of the designed models.  Figure 8 shows the results. Besides, the recognition degrees are compared from five aspects.

After the application, the recognition degree has increased by a certain percentage. The recognition of clear knowledge points has increased from 35% to 42%, and the proportion is not very obvious. Flexibility increases from 21% to 47%, and effective review increases from 24% to 52%. Both have some improvement. The self-learning ability has improved the most, from 18% to 72%. The improvement in learning efficiency has increased from 39% to 82%, a great improvement. Flexibility has also been improved to some extent. As a result, the designed model is helpful to the learning of sports microcourses.

### 5.4. Satisfaction Analysis of Microcourse Teaching Resources

**Figure 9:**
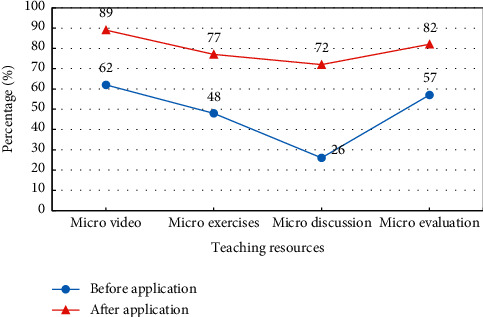
Satisfaction analysis of microcourse teaching resources.

Students' satisfaction with microcourse teaching resources before and after the application of the design model is discussed through microvideos, microexercises, microdiscussions, and microevaluations. The results are shown in Figure 9.

Figure 9 shows that the designed model can improve students' satisfaction with microvideos, from 62% to 89%. Satisfaction with the microexercises increases from 48% to 77%. Microdiscussion satisfaction increases from 26% to 72%. Microevaluation satisfaction increases from 57% to 82%. Satisfaction with all teaching resources of microcourses has been improved. Students are most satisfied with microvideos followed by microevaluations. The designed model stimulates students' interest in learning and improves the resource utilization of students in gymnastics microcourse teaching. It promotes the teaching and reform of gymnastics courses for sports professionals.

## 6. Conclusion

It is significant to study various teaching methods and design teaching modes with the continuous advancement of the reform of university physical education. This design introduces the FC and studies the DL model. Based on this, the DL theory is combined with the microcourse of college sports gymnastics. The reform of the microcourse teaching of physical gymnastics is achieved through the optimization design of the teaching model. The teaching status and the teaching effect after the application of the design model are evaluated and analyzed through questionnaires. The results are as follows: (1) most teachers prefer an interest-oriented teaching philosophy. In the evaluation of students' interest in learning, the proportion of interested students is relatively small, and the proportion of students who are not interested exceeds 50%. (2) The main reasons for the lack of students' interest are the low quality of microvideos and the lack of supervision and reward mechanisms. The main reasons for the low utilization of teaching resources are too long videos and inactive discussion forums. (3) After the application of the design model, the student's interest in learning has been greatly improved, and the proportion of interest has increased to 82.4%. The improvement effect of ordinary college students is the most obvious, with a proportion of 70.8%. (4) The design model can effectively improve the learning effect of microvideo, and the recognition degree of students is significantly increased. Compared with other learning methods, students have the highest recognition. (5) The designed model has the most obvious improvement in improving learning efficiency and autonomous learning ability. The improvement of self-learning ability increases from 18% to 72%. The learning efficiency improvement increases from 39% to 82%. Satisfaction with all teaching resources of microlectures has been improved. The designed model greatly stimulates students' interest in learning and improves resource utilization. It is suggested that schools should strengthen management and improve the mechanism. Educational technology departments should accelerate the realization of breakthroughs in information technology. This design has reference significance for the construction and optimization of microlectures teaching resources based on technical actions. The disadvantage is that only the learning effect of the design model in the gymnastics microcourse is studied, and it is not involved in other courses of the sports major. The research content is narrow. The next step will be to delve into the application of design models in other sports courses and expand the research object.

## Figures and Tables

**Table 1:** Questionnaire distribution and recovery.

Classification	The number of questionnaires issued	Number of returned questionnaires	Number of valid questionnaires	Recovery rate (%)	Questionnaire efficiency (%)
Part 1	130	120	115	92.31	95.83
Part 2	24	24	22	100	96.67
Part 3	100	96	90	96	93.75

**Table 2:** Evaluation of questionnaire validity.

Classification	Very perfect	Perfect	Basically perfect	General	Imperfect
Part 1	4	3	2	1	0
Part 2	3	5	1	1	0
Part 3	5	2	2	1	0

## Data Availability

The experimental data used to support the findings of this study are available from the corresponding author upon request.
